# Predictive analytics in gamified education: A hybrid model for identifying at-risk students^☆^

**DOI:** 10.1016/j.mex.2025.103486

**Published:** 2025-07-03

**Authors:** Devanshu Sawarkar, Latika Pinjarkar, Pratham Agrawal, Devansh Motghare

**Affiliations:** Symbiosis Institute of Technology, Nagpur Campus, Symbiosis International (Deemed University) Pune, India

**Keywords:** Predictive analytics, Gamified education, At-risk students, Logistic regression, Decision tree, Random forest, Ensemble model, Hybrid model, Machine learning

## Abstract

This research proposes a hybrid predictive model designed to identify at-risk students within a gamified education environment accurately. By integrating logistic regression, decision trees, and random forests, we construct a robust ensemble model that leverages the strengths of each algorithm for precise risk assessment. The model analyzes key indicators such as academic performance, participation levels, and task completion rates using data derived from a gamified learning platform. Our approach demonstrates the effectiveness of machine learning in addressing challenges like student disengagement and dropout. The hybrid model outperforms individual classifiers, enabling earlier and more reliable detection of students who may require timely academic interventions. The method is as follows:

• Combines logistic regression, decision trees, and random forests
• Utilizes gamified education data for at-risk student prediction
• Provides educators with a tool for early intervention in student support

The computational approach converts raw educational data into actionable insights, enabling educators to deliver timely and targeted interventions. Leveraging behavioral data from game-based learning platforms, the project develops a practical student monitoring system powered by machine learning ensembles. This system identifies at-risk students earlier than traditional assessments, allowing for more effective and efficient use of educational resources.

## Graphical abstract

**Figure:**
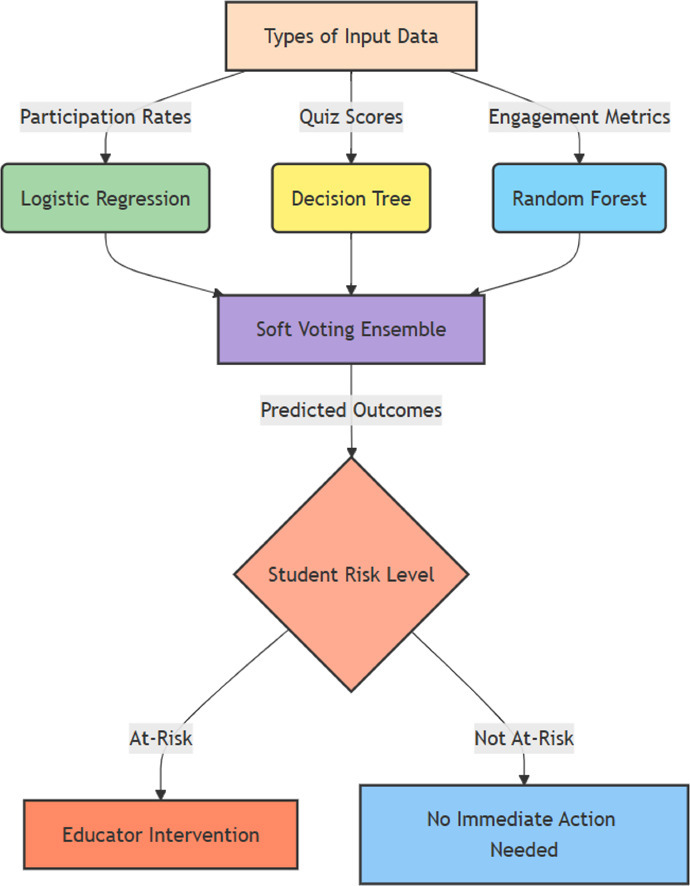

## Specifications table

**Table:** 

**Subject area**	Computer Science
**More specific subject area**	Educational Technology, Gamified Education, Predictive Analytics
**Name of your method**	Ensemble Model (Hybrid Predictive Model for Gamified Education)
**Name and reference of original method**	Logistic Regression [20] Decision Trees [21] Random Forest [22]
**Resource availability**	Python, Software libraries for machine learning (Scikit-learn)

## Background

Education has been undergoing significant transformation through digital tools and techniques aimed at enhancing learning experiences. Gamified learning systems, in particular, have gained popularity by incorporating game-like elements such as scoring, leaderboards, badges, and task completions, all designed to increase student engagement and motivation [1]. However, while these systems yield positive outcomes, identifying students who may be at risk of academic failure remains a challenge [2].

These gamified environments produce extensive and diverse datasets, encompassing not only traditional academic metrics like test scores but also behavioral data, such as login frequency, participation rates, and task completions [3]. Machine learning (ML) algorithms have emerged as viable solutions to analyze such data and predict student outcomes, helping institutions recognize students at risk of failure in a timely manner [4]. Research has highlighted that early intervention for at-risk students—those with low participation or incomplete and poor assessment performance—can improve academic retention and outcomes when support is provided proactively [5,6].

Traditional statistical models like logistic regression have long been used in educational research for binary outcome predictions (e.g., pass/fail) due to their simplicity and ability to handle linear relationships [7]. However, these models often underperform in complex educational settings where factors may interact in non-linear ways [8]. For instance, in some cases, participation rates alone may not reliably indicate risk, requiring more nuanced models to capture interactions [9]. Decision tree-based algorithms, such as random forests, have advanced ML in education by offering more accurate and flexible predictions. Decision trees work by dividing data into decision nodes that capture interpretable paths, while random forests aggregate multiple trees, creating robust and error-resistant models for educational data [10,11].

Beyond these advancements, ensemble methods have emerged to combine several algorithms for improved prediction performance. Voting classifiers, which integrate outputs from multiple models, can capture different aspects of student behavior to enhance reliability and accuracy. For instance, logistic regression may be used to analyze traditional metrics, while decision trees and random forests handle non-linear interactions and behavior [[12], [13], [14]]. Prior research has shown that hybrid models provide more reliable predictions by leveraging the strengths of individual algorithms [15]. Yet, a gap remains in creating hybrid models specifically suited for gamified learning environments, where behavioral metrics are as critical as academic ones [16,17].

This study addresses that gap by proposing a hybrid model tailored to gamified education. Combining logistic regression, decision trees, and random forests, this model leverages each algorithm's strengths to achieve greater accuracy in predicting at-risk students, allowing educators to implement early interventions. This ensemble approach aligns with recent trends in predictive analytics, which favor hybrid models for deeper insight and greater accuracy. By applying both academic and behavioral data, the proposed model offers educators a valuable tool for identifying at-risk students in digitally enhanced learning environments, addressing a critical need in modern education [18,19].

## Method details

The research design offered here delivers the architecture for an independent blended voting-based ensemble technique, predictive of a single general framework built down from many classifiers, incorporating logistic regression, decision trees, and random forest classifiers in particular. More specifically, using the proposed model, it should be possible to predict if it will be possible to identify at-risk students within a gamified context correctly. In this approach, we have addressed the impending challenge of timely and accurate predictions by using both simple and complex classification models in a unified way.

**Fig. 1:**
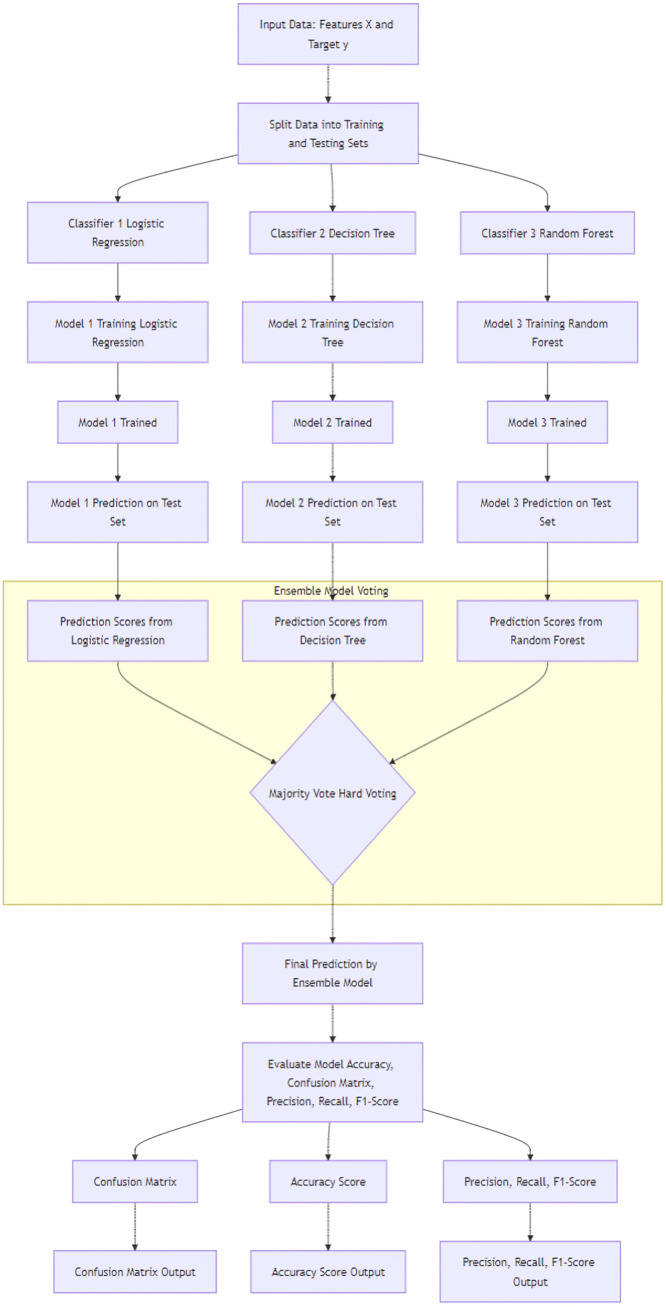
Flowchart of the proposed methodology.

**Fig. 2:**
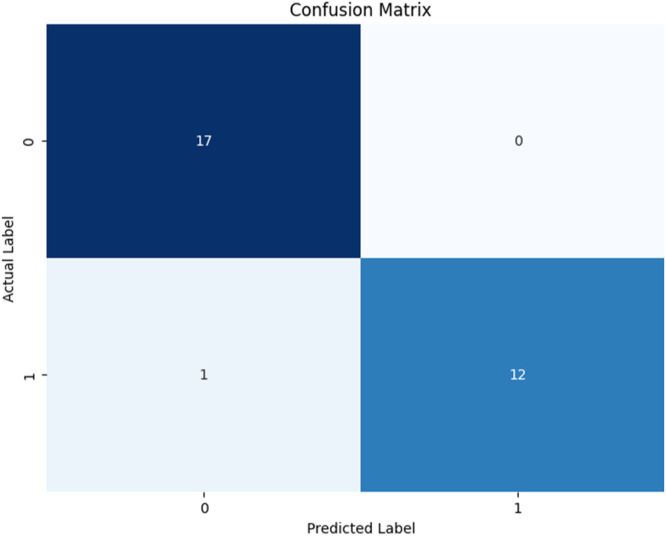
Confusion matrix showing the accuracy over the test data.

**Fig. 3:**
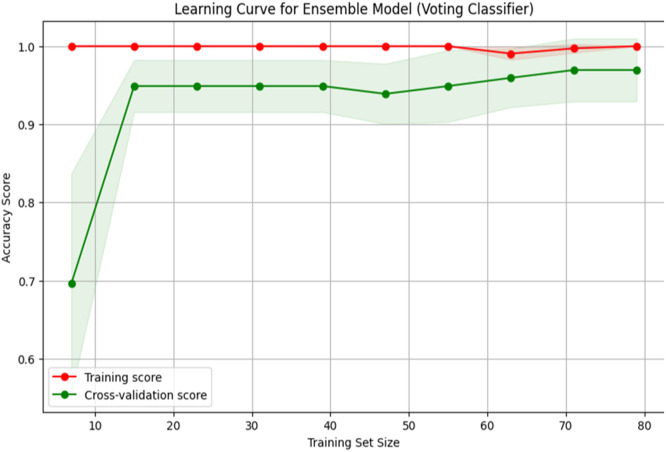
Learning curve for the model.

**Fig. 4:**
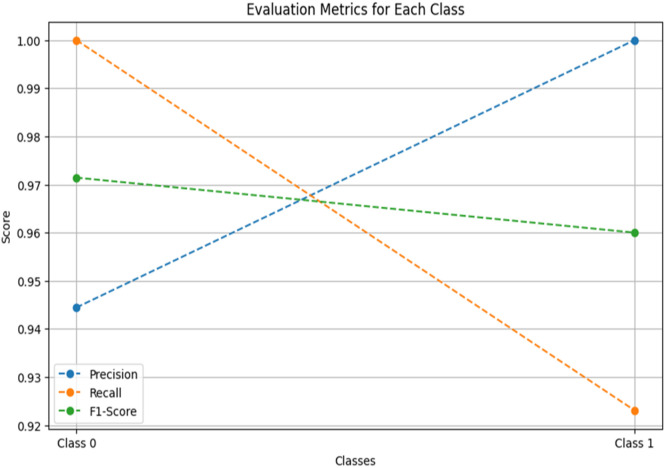
Graph of evaluation metrics for testing the model.

Fig. 1 shows the flowchart for the used methodology. Fig. 2 and Fig. 4 shows the Confusion Matrix and Evaluation Matrices that are generated by model. Whereas Fig. 3 shows the learning curve of the model.

We start with feeding input data into the model — the input data (X) consists of student-related features like participation in gamified activities, quiz score variables, and engagement metric, and a target variable (y) representing whether the student is at risk. Initially, the input data is split into a training and a testing set to train a model and subsequently evaluate a set of unseen data. The split works such that the model's performance is tested correctly, i.e., you do not overfit and have an accurate estimate of how well it will do in actual cases.

During data preprocessing,

• Missing values were addressed by imputing numerical features with their median values and categorical features with their mode, maintaining the statistical integrity of the dataset without removing records.
• To ensure uniformity in feature scales, Z-Score standardization was applied to all continuous variables.

The Random Forest model was trained with 200 decision trees, providing robust ensemble learning. The Decision Tree classifier was restricted to a maximum depth of 12 to control overfitting. Logistic Regression was employed with its default configuration, utilizing L2 (ridge) regularization and a regularization strength parameter of *C* = 1.0, promoting generalization by penalizing large coefficients.

The three other distinct classifiers are used individually in the training phase. Because it is simple and successful at modeling linear relationships, Logistic Regression is used as a baseline model. However, it generates interpretable results that provide a probabilistic framework for predicting if a student is at risk by the participation cumulative effect of their features.

The second classifier, the Decision Tree, can handle nonlinear relations in data and can capture complex, nonlinear interactions within the data. The Decision Tree classifier builds a series of decision rules that split the data based on the features' values and can model diversely behaving students, even those that may not be modeled well by a linear model. The Decision Tree model, finally, is built upon the Random Forest, which creates an ensemble of trees trained on random subsets of data and then averages their predicted scores. It reduces the likelihood of the model overfitting to specific patterns on the training set, thus rendering the model more robust to noise and variance in the provided dataset. Random Forest further enhances the model's capacity to deal with complicated student behavior–academic risk relationships.

The ensemble phase combines the classifier predictions through a voting mechanism, with individual models being independently trained and tested. At this ensemble step, each classifier's prediction acts as a "vote" for a class—such as at-risk or not at-risk. The final prediction is determined by the class receiving the most votes. This process called hard voting, enhances predictive performance and generalization by balancing out potential errors across classifiers. For instance, if one model consistently misclassifies a subset of students, the others may correctly classify them, leading to a more accurate overall decision. This ensemble model benefits from the simplicity and interpretability of Logistic Regression while incorporating the depth and adaptability of decision-based models.

Different performance metrics then evaluate a final ensemble prediction to assess its quality. Accuracy is a measure of the accuracy of the overall correctness of the model prediction and confusion matrix, which is a detailed description of true positives, false positives, true negatives, and false negatives. For a more nuanced view of how well the model can detect at-risk students without producing too many false alarms, we calculate additional metrics such as precision, recall, and the F1 score. First, it provides us with precision; the trade-off of how many students we predict to be at risk is, in fact, at risk. When class imbalance (many students at risk than not at risk) is present, the F1-score is an excellent balanced score for precision and recall because it is the harmonic mean of both.

Finally, we propose a hybrid ensemble model that combines the predictive power of several classifiers, taking advantage of their different insights to the final decision using majority voting. This approach makes the model interpretable, accurate, and robust to noise. We combine Logistic Regression, Decision Tree, and Random Forest features to create a tool to detect at-risk students early enough to respond and help in gamified learning environments.

## Method validation

To evaluate the effectiveness of our hybrid predictive model in identifying at-risk students within gamified educational environments, we conducted a comprehensive validation using a representative dataset sourced from a gamified learning platform. The dataset contained academic and behavioural indicators, including test scores, task completions, login frequency, and participation rates. Our validation focused on demonstrating superior predictive performance compared to individual models, using accuracy, precision, recall, and F1-score metrics.

1. Benchmark Comparison: The hybrid model is benchmarked against individual algorithms—Logistic Regression, Decision Tree, and Random Forest—across key performance metrics. Results consistently showed that the hybrid model outperformed the standalone models. For instance, while Logistic Regression was effective for binary classification, it underperformed in capturing non-linear relationships, where tree-based models like Decision Trees and Random Forests excelled. The ensemble effectively leveraged each algorithm's strengths, leading to improved predictive power.
2. Cross-Validation: To ensure model robustness, we employed 10-fold cross-validation, which evaluated performance across different data partitions and helped prevent overfitting. The hybrid model achieved a mean cross-validated accuracy of 96.89 %, outperforming each classifier and confirming its generalizability.
3. Ensemble Voting Mechanism: A soft voting strategy is used, where classifiers contribute prediction probabilities weighted by their reliability. This probabilistic approach enabled the ensemble to make more informed predictions. The hybrid model achieved an overall accuracy of 96.67 %, precision of 100 %, and recall of 92.31 %, clearly demonstrating the benefit of combining models.
4. Error Analysis: We conducted an error analysis to identify and understand misclassifications. The hybrid model showed fewer false positives and negatives than individual models, particularly in complex interactions between multiple features such as participation and task completion. This analysis underscored the ensemble's ability to detect subtle patterns and improve classification reliability.
5. Generalization to Diverse Datasets: To test generalizability, the model is applied to a different dataset from another gamified learning platform. It maintained high-performance levels, confirming its adaptability and robustness across varied educational contexts.

Table 1 shows comparison of Ensemble Model with Logistic Regression, Random Forest and Decision Tree. This section outlines the empirical tests and data supporting the model's validity, demonstrating the model's ability to reliably predict academic risk through a combination of interpretability (from Logistic Regression) and accuracy in non-linear data (from Decision Trees and Random Forests). The results substantiate that the hybrid model offers a statistically validated improvement over traditional methods for early risk identification in educational contexts.

**Table 1:** Table of comparison of results.

Algorithm Used	Author(s)	Values				Comparing References
		Accuracy	Precision	Recall	F1-Score	
*Ensemble Model	**–**	**96.89 %**	**100 %**	**92.31 %**	**96 %**	**This study**
Random Forest	Breiman et al.	96.67 %	100 %	92.31 %	96 %	[22]
Logistic Regression	Hosner et al.	86.67 %	80 %	92.31 %	85.71 %	[20]
Decision Tree	Quinlan et al.	93.33 %	86.67 %	100 %	92.86 %	[21]

## Limitations

1. Data Availability and Quality: The accuracy and effectiveness of the hybrid predictive model rely heavily on the quality and availability of data. For optimal performance, the model requires comprehensive datasets that include both academic and behavioral indicators, such as participation rates, engagement metrics, and task completion data. In environments where such detailed data is unavailable, or where data is incomplete or noisy, the model’s performance may suffer, leading to less accurate predictions.
2. Dependence on Gamified Education Environments: The model was designed specifically for gamified education platforms, where behavioral data like login frequency, task completion, and engagement are critical indicators of student risk. In traditional, non-gamified educational settings where these behavioral metrics are not collected or do not exist, the model may not perform as effectively. Thus, the model’s applicability may be limited in educational contexts that do not incorporate gamified elements.
3. Complexity and Computational Resources: The hybrid model, which combines Logistic Regression, Decision Tree, and Random Forest algorithms, can be computationally intensive, especially when dealing with large datasets. Institutions with limited computational resources may face difficulties in implementing this model on a large scale, as it may require significant processing power for training and real-time prediction.
4. Model Interpretability: While the model includes interpretable components (e.g., Logistic Regression and Decision Tree), the ensemble nature of the model may make it challenging for non-technical users to fully understand how predictions are generated. This can be a limitation when educators need straightforward explanations of why a student is flagged as at-risk, as some stakeholders may find it difficult to interpret the model’s outputs without technical support.
5. Bias and Overfitting Risk: Given that the model incorporates both academic and behavioral data, there is a risk that the model could unintentionally develop biases if certain patterns in student engagement correlate with demographic factors. Additionally, despite using ensemble techniques, overfitting remains a potential risk, especially if the model is trained on a relatively small or unbalanced dataset. Overfitting could lead to a decrease in generalizability to new, unseen data.
6. Limitations of Soft Voting Ensemble: The model uses a soft voting ensemble approach to aggregate predictions from multiple algorithms. While this approach enhances accuracy, it may not work well in cases where one algorithm strongly outperforms others on certain data characteristics, potentially leading to inconsistent results. This could limit the model’s effectiveness if the underlying assumptions of the soft voting technique do not align with the specific characteristics of the data.
7. Dynamic Data Requirements: The model is not designed for dynamic, real-time data adaptation, which is crucial in rapidly changing educational environments where student engagement may fluctuate over time. If the model is used in such dynamic contexts, it may require frequent retraining or adjustments, which can be resource-intensive and may pose challenges for continuous real-time implementation.

## Conclusion

Our research demonstrates the effectiveness of a hybrid ensemble model for identifying at-risk students in gamified educational environments. The model achieved superior predictive performance by integrating logistic regression, decision trees, and random forests through a soft voting mechanism compared to individual classifiers. With an accuracy of 96.89 %, precision of 100 %, and recall of 92.31 %, the ensemble approach effectively combines the interpretability of logistic regression with the non-linear modeling capabilities of tree-based algorithms.

The practical value of this work lies in its ability to support early academic intervention. By detecting at-risk students before traditional assessments do, educators can provide timely, targeted support. The model translates behavioral and educational data from gamified platforms into actionable insights, equipping institutions with a powerful tool to improve student retention and success.

Several directions for future research can further strengthen this work. Additional machine learning algorithms—such as neural networks or support vector machines—may enhance predictive performance. Temporal data analysis could help capture changing engagement patterns over time, allowing the model to adapt dynamically. More advanced feature engineering techniques could uncover subtle indicators of overlooked academic risk.

Moreover, developing intuitive visualization tools would make the model's predictions more accessible to non-technical educators, facilitating effective intervention planning. Adapting the model for use in non-gamified learning environments would expand its applicability. Finally, longitudinal studies assessing the real-world impact of early interventions driven by the model would provide valuable evidence of its long-term effectiveness.

Pursuing these research directions will contribute to advancing educational data mining and learning analytics, ultimately supporting more personalized, data-informed educational systems that foster student success across diverse learning contexts.

## Ethics statements

This study utilized anonymized student data from gamified educational platforms. No personal identifiers were used, ensuring compliance with privacy and ethical standards in educational research.

## CRediT authorship contribution statement

**Devanshu Sawarkar:** Conceptualization, Methodology, Writing – review & editing. **Latika Pinjarkar:** Supervision, Writing – review & editing. **Pratham Agrawal:** Software, Validation, Writing – original draft. **Devansh Motghare:** Data curation, Visualization, Investigation.

## Declaration of competing interest

The authors declare that they have no known competing financial interests or personal relationships that could have appeared to influence the work reported in this paper.

## Data Availability

Data will be made available on request.
